# Technological Potential of *Lactobacillus* Strains Isolated from Fermented Green Olives: *In Vitro* Studies with Emphasis on Oleuropein-Degrading Capability

**DOI:** 10.1155/2016/1917592

**Published:** 2016-06-30

**Authors:** Massimo Iorizzo, Silvia Jane Lombardi, Vincenzo Macciola, Bruno Testa, Giuseppe Lustrato, Francesco Lopez, Antonella De Leonardis

**Affiliations:** ^1^Department of Agriculture, Environmental and Food Sciences (DiAAA), University of Molise, Via De Sanctis, 86100 Campobasso, Italy; ^2^Department of Biosciences and Territory (DiBT), University of Molise, Contrada Lappone, Pesche, 86090 Isernia, Italy

## Abstract

Technological properties of two strains of* Lactobacillus plantarum* (B3 and B11) and one of* Lactobacillus pentosus* (B4), previously isolated from natural fermented green olives, have been studied* in vitro*. Acidifying ability, salt, temperature, and pH tolerances of all strains were found in the range reported for similar strains produced in Italy and optimal growth conditions were found to be 6.0–8.0 pH, 15–30°C temperature, and less than 6% NaCl. Moreover, all strains showed very good tolerance to common olive phenol content (0.3% total phenol) and high oleuropein-degrading capability. It was found that medium composition affected the bacterial oleuropein degradation. B11 strain grown in a nutrient-rich medium showed a lower oleuropein-degrading action than when it was cultivated in nutrient-poor medium. Furthermore, enzymatic activity assays revealed that oleuropein depletion did not correspond to an increase of hydroxytyrosol, evidencing that bacterial strains could efficiently degrade oleuropein via a mechanism different from hydrolysis.

## 1. Introduction

Table olives are a very popular fermented vegetable, consumed all over the world and produced principally in Spain, Italy, and Greece [1]. Olive is the fruit of the domestic olive tree (*Olea europaea* L.); it is botanically a drupe formed by a thin membranous outer layer (epicarp), a fleshy mesocarp, and a stony endocarp. Unripe olives are not edible due to a high oleuropein content that gives an intense bitter taste. However, several processes can make drupes edible. “Spanish-Sevillian method” for green olives, “Californian method” for black oxidized olives, and “Greek method” for naturally black olives are the prevalent industrial processes. However, several traditional protocols have been taken into consideration depending on the olive maturation extent (green or black olives) and on the fermentation conditions [2].

Oleuropein degradation and fermentation are the most important steps in the production of the green table olives. Oleuropein is a *β*-glucoside, present in the green olives up to 1-2%, which naturally declines during maturation [3]. Total or partial depletion of oleuropein is fundamental to make olives suitable for human consumption. According to the “Spanish-Sevillian” method oleuropein is chemically hydrolyzed by treating the green olives with diluted sodium hydroxide solution [4]. Conversely, in the so-called “natural olive,” the fruits are directly brined. In this case, olive debittering occurs very slowly and the oleuropein disappears in the fruits as result of its enzymatic and microbial hydrolyses and for diffusion from the pulp to the brine [5–7].

Olive fermentation is fundamental for the production of high-quality product. Principally, microorganisms metabolize the sugars contained in the olive mesocarp producing lactic acid. The low pH of the brine, together with the production of antimicrobial substances, minimizes undesirable microorganism population and increases safety of product [8]. Generally, a spontaneous fermentation is performed by the indigenous microbiota present in both the raw material and the processing environment [9, 10]. Indigenous microbiota varies with the quality of drupes, harvesting conditions, and postharvesting treatments and it is composed mostly of lactic acid bacteria (LAB) and yeasts. However, several studies have evidenced that* Lactobacillus plantarum* and* Lactobacillus pentosus* are the chief species in the spontaneous fermentation process [9, 11].

Generally, fermentation is carried out during the olive manufacture by an empirical process and use of starter cultures is still not very common, although this could ensure an improved and standardized product [12]. With this aim, many efforts have been addressed to isolate wild* Lactobacillus* strains exhibiting good technological potential in the production of table olives [13]. Selection of starter cultures is a laborious progress and several aspects have been investigated, such as adaptation to a particular substrate, growth data, acidifying capacity, salt and pH tolerance, temperature range, flavour, and bacteriocin production [14]; moreover, probiotic potential and oleuropein-degrading capability are other key requirements [15, 16].

Cultures with oleuropein-degrading capability could be useful to remove fruit bitterness or to reduce the time necessary for olive debittering route. Actually, although this approach has been applied widely for black olives, it has not been used likewise for green olives [17, 18]. Nevertheless, microbial debittering of green olives as a possible alternative to lye treatment is an interesting biotechnological innovation that could meet positively the rising demand of the modern customer for naturally/minimally treated foods and more environmentally friendly processes [19].

In a previous study an oleuropein drop of almost 85% (after only seven days) has been obtained in green olives naturally fermented in a broth containing 0.3% glucose and 0.05% yeast extract in water [20]. By considering this promising result, molecularly identified* Lactobacillus* strains were isolated from the abovementioned broth and three of them were characterized in the present work. Specifically, growth data, acidifying ability, enzymatic activities, and oleuropein-degrading capability of the LAB were investigated* in vitro* under different conditions.

## 2. Materials and Methods

### 2.1. Chemicals, Bacterial Strains, and Culture Media

All reagents were of analytical grade and unless otherwise stated were purchased from Sigma-Aldrich Co. (St. Louis, MO, USA). Olive leaf liquid complex (OL), produced by Barlean's Co (Ferndale, WA, USA), was purchased in a commercial store. This product contains 4% glucose and a mix of the principal olive fruit phenols, including oleuropein, verbascoside, hydroxytyrosol, tyrosol, caffeic acid, and others. Total phenol in OL was 9.0 mg/mL as GAE (gallic acid equivalent), and oleuropein was estimated to be around 62% of total phenol.

Bacterial strains were isolated from cv. “*Nocellara del Belice*” green olives naturally fermented as described in De Leonardis et al. [20] and phenotypically and genetically characterized according to Testa et al. [21]. The strains, used in this study, have been identified on the basis of sequencing as* Lactobacillus plantarum* (B3 and B11) and* Lactobacillus pentosus* (B4) as indicated in Table 1.

Culture media were prepared in the laboratory as follows: (i) modified MRS broth containing 1% glucose (MRSm); (ii) MRSm mixed with OL (3 : 1, v/v) (MRSm-OL); (iii) nutrient water broth containing 0.3% glucose and 0.05% yeast extract in water (NWB); (iv) NWB prepared in 0.1 M citrate-phosphate pH 5.0 (NWB-pH 5). All media were autoclaved at 121°C for 15 min.

### 2.2. Experimental Plan

MRSm, MRSm-OL, and NWB were used to determine the bacterial viability and the fermentative metabolism by monitoring, at 30°C up to 15 days, the total bacterial count, glucose, and pH, respectively. MRSm was used to assay the bacterial growth at different pH (3, 6, and 8 values, adjusted before sterilization with HCl or NaOH 1 N), incubation temperatures (7, 15, and 30°C), and salt concentration (4, 6, and 10% NaCl). In this case, the increase of optical density at 660 nm (OD 660 nm) and the change of pH after 0, 1, and 7 days were measured. The OD 660 nm of MRSm was used as blank.

### 2.3. Analytical Determinations

Glucose was determined by a specific enzymatic kit purchased from Steroglass (Perugia, Italia). Total phenol content was determined with the Folin-Ciocalteu's method by using an independent gallic acid calibration curve as reference. HPLC analysis was performed with a Varian ProStar 230 instrument (Mulgrave, AUS), equipped with a column Kinetex 5u C18 100Å (150 × 4.6 mm) (Phenomenex, USA) and supplied with UV-Vis detector set up at a wavelength of 240 and 280 nm. Chromatographic separation was carried out according to the conditions reported in Cuomo and coworkers [22]. Oleuropein (PubChem CID: 5281544) and hydroxytyrosol (PubChem CID: 82755) were quantified through a corresponding calibration curve derived from a plot of area counts versus concentration.

### 2.4. Multienzymatic Activities

Enzymatic activities were determined using the Api-Zym system (BioMérieux Italia, Rome). Rapid semiquantitative evaluation of 19 hydrolytic enzymes was carried out [23], each gallery was inoculated with 165 *μ*L of suspension, and, after incubation at 37°C for 4 h, one drop of each of the reagents Zym A and Zym B was added. The color that developed in each enzymatic reaction was graded from (+) positive to (−) negative and (W) weakly positive by the Api-Zym color reaction chart.

### 2.5. Oleuropein-Degrading Capability and *β*-Glucosidase Activity

Each selected strain (B3, B4, and B11) was inoculated (10^8^ CFU/mL) in NWB-pH 5 broth supplemented with 0.5 mg/mL oleuropein and incubated at 30°C. Samples containing only oleuropein solution without inoculum were also analyzed for comparison (control). At fixed times (24, 48, and 72 hours) 0.25 mL of sample was taken from each tube, filtered through a 0.45 *μ*m PVDF filter, and directly injected to the HPLC for monitoring the concentration of oleuropein and hydroxytyrosol.

In parallel and independent tests, the oleuropein-degrading capability and *β*-glucosidase activity of a* L. plantarum* B11 cell suspension were evaluated. In this case, for the oleuropein-degrading capability, reaction mixtures contained 0.5 mL oleuropein (0.5 mg/mL) or OL solution (0.2 mg/mL as GAE), 1 mL cells (10^8^ CFU/mL), and 2 mL citrate buffer. After 4 and 18 hour, 0.25 mL of reaction solution was treated with methanol (0.5 mL); after centrifugation, the supernatants were evaporated by rotary-evaporator recovering the residue in 0.25 mL water before the HPLC analysis. Instead, for the *β*-glucosidase activity the absorbance at 405 nm related to the amount of p-nitrophenol (p-NP) (PubChem CID: 980) liberated from p-nitrophenyl-*β*-D-glucopyranoside (p-NPG) (PubChem CID: 259380) was monitored using a Varian spectrophotometer equipped with thermostatted cell holder. p-NP esters are used to assay various enzymatic activities [24–26]. Reaction mixtures contained 50 *μ*L p-NPG (1 mg/mL), 100 *μ*L cells (10^7^ CFU/mL), and 350 *μ*L citrate buffer. At predetermined time intervals (2, 4, 6, and 18 h), the reaction was stopped by the addition of 500 *μ*L of cold 0.2 M sodium carbonate (4°C). Samples were centrifuged and the supernatants were collected for UV-Vis analysis.

### 2.6. Statistical Analysis

All analytical assays were carried out in three replicates by determining the mean and standard deviation. Statistical analyses were performed by using SPSS 13.0 software (SPSS, Inc., Chicago, IL, USA). Significant difference was evaluated using ANOVA LSD test at *p* < 0.01.

## 3. Results and Discussion

### 3.1. Bacterial Viability and Fermentative Metabolism in Different Culture Media

**Figure 1:**
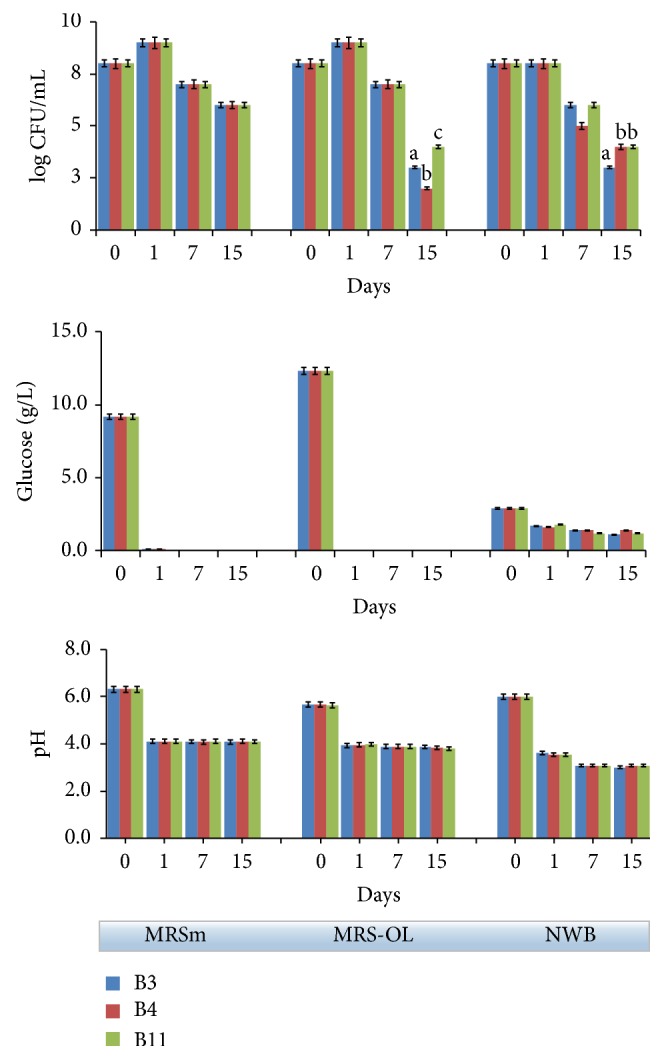
Bacterial growth, glucose, and pH evolution followed up to 15 days in different culture media (MRSm: modified MRS containing 1% glucose; MRS-OL: MRSm supplemented with olive leaf liquid complex; NWB: nutrient water broth containing 0.3% glucose and 0.05% yeast extract, in water) inoculated separately with the* L. plantarum* (B3, B11) and* L. pentosus* (B4) strains and incubated at 30°C. Data represent the mean and standard deviation of three independent replicates. Only the differences resulting as statistically significant (*p* < 0.01) are indicated with different letters.

In Figure 1 the cell viability and fermentative metabolism of the three strains is shown (B3, B4, and B11) in different culture media.

As shown, very similar behavior between strains was found. The commonly used MRS was prepared with a content of glucose 1%, w/v (MRSm) by considering that olives contain low amounts of sugar. In the MRSm, all the strains showed a very high acidifying ability, degrading all glucose in 24 hours with final pH values of about 4.1. Furthermore, even in the absence of glucose a high cell viability was observed up to 15 days.

A commercial preparation of olive leaves (OL), sold commonly as a dietary supplement, was used in order to investigate influence of phenols on strain viability. Preliminarily, OL was used in the MIC (Minimum Inhibitory Concentration) test evidencing that the selected strains do not show inhibition of cell viability at a concentration of phenols between 0.9 and 9.0 mg/mL (data not shown). Optimal tolerance of the strains to phenol compounds was in accordance with that observed for other* Lactobacillus* strains [27]. In this regard, Landete and coworkers [28] have evidenced that oleuropein, hydroxytyrosol, tyrosol, vanillic,* p*-hydroxybenzoic, sinapic, syringic, protocatechuic, and cinnamic acids did not inhibit the* L. plantarum* growth at the concentration generally found in the olives. However, there are evidences that antibacterial effects of the olive phenolic compounds depend strongly on method used to test them [27].

In this study, cell viability (Figure 1) was tested in MRSm supplemented with OL (MRS-OL) at 0.3% total phenol concentration (as GAE), aiming to simulate the amount of total phenols normally occurring in green olive fruits. After 24 hours, glucose was consumed fully, while pH achieved the minimal value at 3.9. In the subsequent days, bacterial counts progressively decreased, reducing drastically at the 15th day. This last result was similar to what has been observed by Medina et al. [29] who proved few oleuropein derivates; in particular the dialdehydic form of decarboxymethyl elenolic acid, either free (EDA) or linked to hydroxytyrosol (HyEDA), may exhibit an antimicrobial effect against the LAB. However, also the fast loss of glucose may have contributed to the bacterial decline in the MRS-OL samples. Finally, in the NWB samples, in the first 24 hours, glucose was metabolized partially and pH achieved a very low value (about 3.6) (Figure 1). This low pH value brought substantially the end of growth of the strains. Indeed, only insignificant changes of glucose content were found in the subsequent 7 and 15 days, while the final pH was fixed to 3.0. Evidently, the growth on NWB resulted in a sharp drop in pH because the medium was not buffered. However, it should be also outlined that when the fermentation has been carried out in presence of olives, the pH value of SB medium was never found below 4.0 [20].

### 3.2. Growth Data and Acidifying Ability

**Figure 2:**
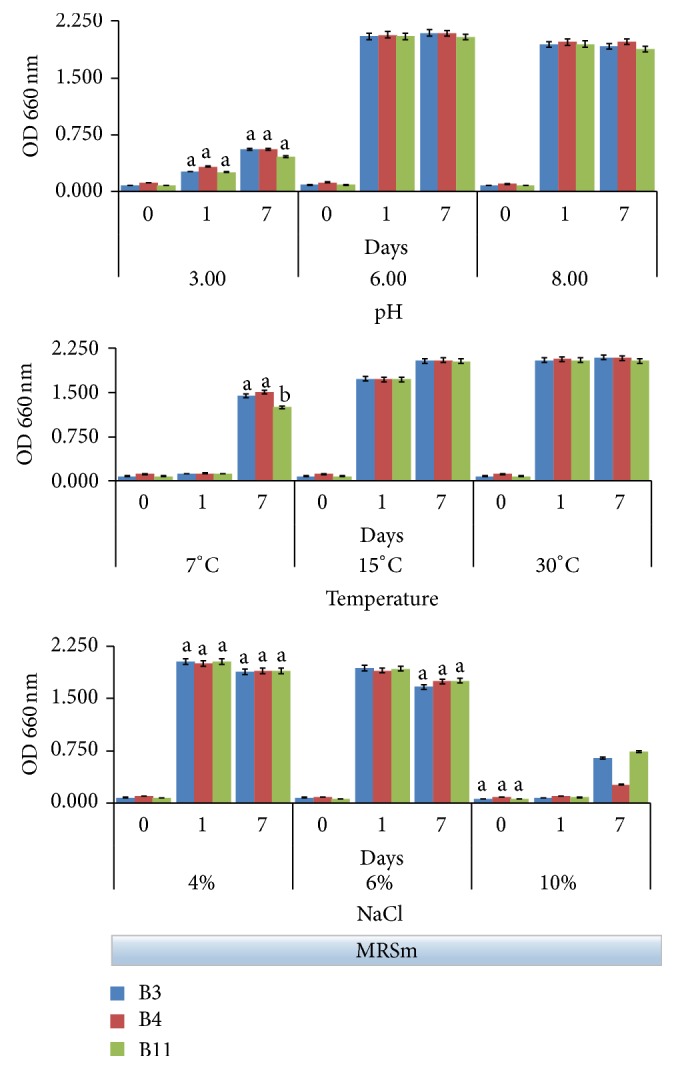
Bacterial growth (OD at 660 nm) in MRSm (modified MRS containing 1% glucose) followed up to 7 days under the following fermentation conditions: pH (3.0, 6.0, and 8.0), temperature (7, 15, and 30°C), and NaCl concentration (4, 6, and 10%). Data represent the mean and standard deviation of three independent replicates. Only the differences resulting as statistically significant (*p* < 0.01) are indicated with different letters. 30°C incubation temperature; initial pH 6.

**Figure 3:**
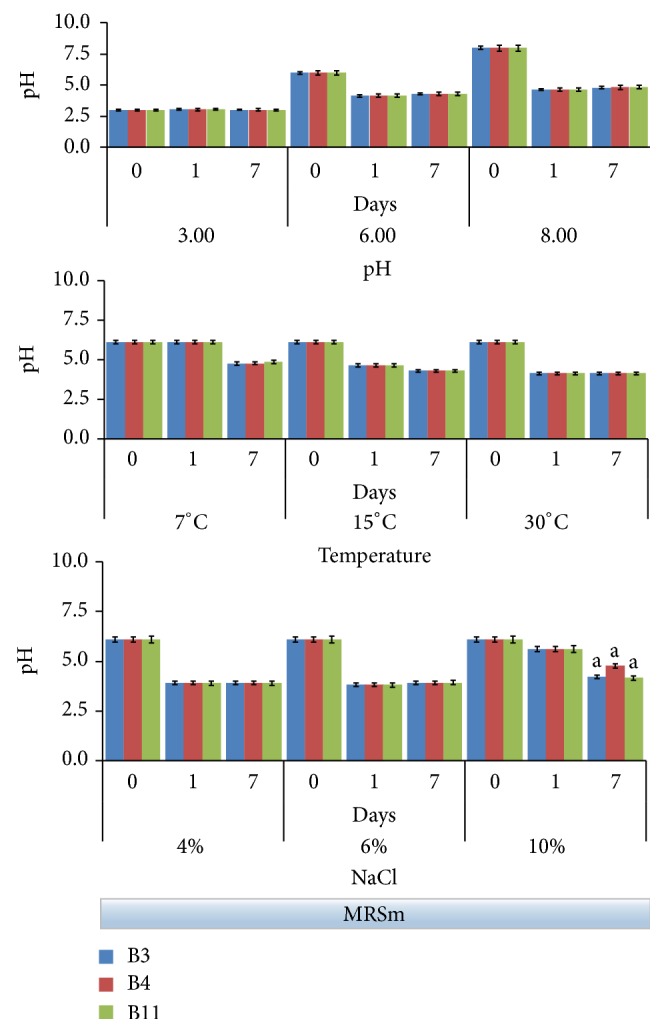
pH evolution in MRSm (modified MRS containing 1% glucose) followed up to 7 days under the following fermentation conditions: pH (3.0, 6.0, and 8.0), temperature (7, 15, and 30°C) and NaCl concentration (4, 6, and 10%). Data represent the mean and standard deviation of three independent replicates. Only the differences resulting as statistically significant (*p* < 0.01) are indicated with different letters. 30°C incubation temperature; initial pH 6.

Both the growth data and the acidifying ability have been tested on B3, B4, and B11 strains under different conditions of pH (3.0, 6.0, and 8.0), temperature (7, 15, and 30°C), and NaCl concentrations (4, 6, and 10%). Bacterial growth was evaluated following the increase of optical density at 660 nm (Figure 2), while acidifying activity was monitored with pH (Figure 3). Observations were stopped after 7 days because this was the time in which the major evidences happened in experimental models with olives, as reported in the our previous work [20].

Both Figures 2 and 3 evidenced that behaviors of the three selected strains were overall very similar to one another.

Generally, the starting brine pH ranged between 5.0 and 6.0 when untreated olives were immersed in water [30], while pH of NaOH debittered olives is typically alkaline and ranged between 8.0 and 9.0 [31]. Similarly, with other* Lactobacillus* strains isolated from table olives [32], selected strains demonstrated that subneutral or alkaline pH did not affect significantly their growth (Figure 2). Conversely, a drastic inhibition was observed at pH 3 followed by a very weak rising after 7 days. In accordance with data shown in Figure 1, a value of pH around 3.0 revealed to be a limiting factor for these strains. Undoubtedly, best growth occurred at pH 6.0, although at pH 8.0 the strain growth was just slightly lower. In these conditions, all strains showed a good acidifying activity, bringing pH in the 4.1–4.6 range already after 24 hours (Figure 3); for the same samples, the pH remained substantially unchanged after 7 days of incubation.

Concerning the ability to grow at different temperatures, the strains grew well at 15 and 30°C (Figure 2). This evidence is in agreement with Aponte and coworkers [11] that showed that 70% of the 88 strains isolated from an Italian olive cultivar and belonging to the* L. plantarum* group exhibited a proper growth at 15°C. At 7°C the strain growth in the first 24 h noticeably slowed down; however, all strains showed a good adaptability to low temperature at the final time (7 days), when the minimal value of pH (4.8 on average) was achieved (Figure 3). Ability to grow at low temperature certainly is a good requisite by considering that temperature during the table olive fermentation generally is not controlled and it can vary according to environmental conditions [12].

In the table olive making, the salt tolerance is one of the most important technological characteristics for starter cultures. High salt tolerance of the* L. plantarum* is well known [33]. Usually, brines are used with NaCl concentrations higher than 6%. The growth of the selected strains was not significantly affected by a concentrations of salt less than 6%, while a drastic reduction at 10% NaCl has been observed (Figure 2). However, both the* L. plantarum* B3 and B11 strains, unlike the* L. pentosus* B4, showed a noticeable recovery of growth at the 7th day evidencing a good adaptability to high salt concentrations. Growth data in presence of salt affected the acidifying activity (Figure 3). In both the 4% and 6% NaCl tests, the pH was less than 4.0 (3.8 on average) for all strains already after 24 h. Similar acid pH value was achieved also in the 10% NaCl test, though more slowly (Figure 3).

### 3.3. Multienzymatic Activities

Enzyme profiling is an important factor for the selection of starter strains because it gives the enzymatic potential of bacteria relative to their technical performance and influence on organoleptic characteristic of the final product [34]. In this study, a pool of enzymatic activity was detected by using the Api-Zym system with aim of displaying enzyme activities of the selected strains. Results are shown in Table 2.

In general, the strains evidenced a very similar behavior to one another. Degradation of amino acids into volatile molecules could play a significant role in the flavour of the table olives. Nevertheless, absence of proteolytic activity could be also a positive characteristic due to protein hydrolysis and further degradation may generate off-flavours and toxic compounds such as biogenic amines [35]. In this regard, the tested strains exhibited positive activity for leucine arylamidase and valine arylamidase and negative activity for cystine arylamidase, trypsin, and *α*-chymotrypsin, respectively. Moreover, all strains did not produce the undesirable *β*-glucuronidase activity [34].

None of three strains showed lipolytic activity since esterase (C4), esterase lipase (C8), and lipase (C14) activities were not detected.

Acid phosphatase and naphthol-AS-BI-phosphohydrolase activities, involved in phytic acid degradation, were found to be weakly positive and positive, respectively. Phytase activity is considered desirable characteristic of starter cultures for production of several fermented vegetable [36]. Concerning the activities of enzymes correlated with carbohydrate catabolism, *α*-galactosidase, *α*-mannosidase, and *α*-fucosidase were no detected, while *α*- and *β*-glucosidase activities were found in all tested strains. Otherwise, B3 and B4 strains have shown higher *β*-galactosidase activity in respect to B11. *β*-galactosidase activity could increase the potential use of the tested LAB strains as starter and probiotic organisms [37].

Finally, all strains exhibited positive a- and b-glucosidase activities and this feature could play a determinant role for oleuropein hydrolysis [38, 39]. The *β*-glucosidase hydrolyzes glucose forming oleuropein aglycon derivates, while the esterase hydrolyzes the ester bonds of oleuropein, producing glucosyl derivates, hydroxytyrosol, and elenolic acid. As said above, all tested strains exhibited *β*-glucosidase activity, while they did not produce esterase.

### 3.4. Oleuropein-Degrading Capability and *β*-Glucosidase Activity

Evolution of oleuropein and hydroxytyrosol during olive fermentation was widely studied. Degradation of oleuropein is attributed mainly to *β*-glucosidase hydrolytic activity of the microorganisms that metabolize the free glucose, releasing the hydroxytyrosol as waste product [27, 28]. Indeed, hydroxytyrosol increases during the fermentation, remaining one of the principal phenols found in the table olives [40]. Nevertheless, by considering relevant literature data, it should be reported that hydroxytyrosol is produced gradually and in nonstoichiometric amounts with oleuropein decrease [7, 20, 32, 38].

Oleuropein and hydroxytyrosol have been determined in the MRS-OL samples at the end of the incubation period (15 days) and the results were compared with the values found in the corresponding controls without bacteria (data not shown). Oleuropein depletion was not completed and actually, only by 25–30% oleuropein decrease was observed. On the other hand, an increase of hydroxytyrosol ranging from 52% (B3 strain) to about 27% (B4 and B11 strains) was found. These results were markedly below expectations.

**Figure 4:**
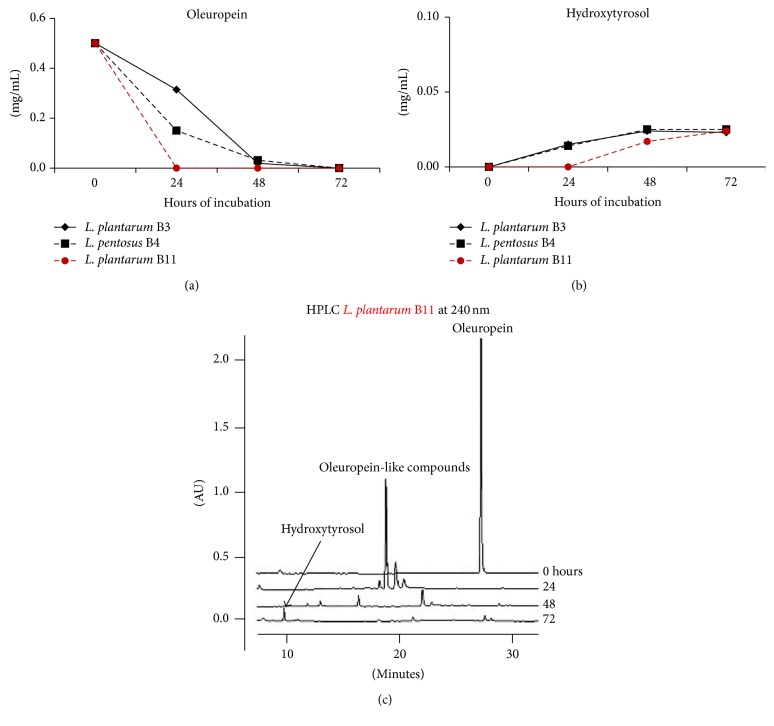
Changes of oleuropein (a) and hydroxytyrosol (b) followed up to 72 hours in NWB-pH 5, inoculated with the* L. plantarum* (B3, B11) and* L. pentosus* (B4) strains and incubated at 30°C. In addition, HPLC chromatograms relative to B11 strain are shown (c). Data represents the mean of three independent replicates.

Ruiz-Barba and coworkers [41] highlighted that presence of organic compounds (amino acids and proteins) in the assay medium could modify interactions between bacteria and phenolic compounds. For this reason, a poor nutrient medium (NWB-pH 5), buffered at pH 5 and supplemented with pure oleuropein (0.50 mg/mL), was preferred to study the oleuropein-degrading capability of the LAB strains (Figure 4).

In this culture medium the B11 showed the best performance by degrading all oleuropein already after 24 hours (Figure 4(a)), while oleuropein depletion was complete after 72 hours for the other two strains (B3 and B4).

Degradation of oleuropein did not match a significant increase of hydroxytyrosol (Figure 4(b)), which was produced in very low amounts (approximately less than 0.03 mg/mL in 72 hours). Absence of free hydroxytyrosol leads one to suppose that selected strains could degrade oleuropein via a different mechanism. Actually, oleuropein degradation is not exclusively linked to *β*-glucosidase and esterase due to the fact that also polyphenoloxidase and peroxidase have been reported to catalyze oleuropein transformations [7]. Specifically, polyphenoloxidase has been found to be more effective in degrading oleuropein than hydrolyzing enzymes [38]. HPLC chromatograms relative to the activity of the B11 (Figure 4(c)) evidenced the formation of intermediate compounds having a peak of absorbance at 240 nm typically related to oleuropein derivates, in accordance with literature data [28, 29].

**Figure 5:**
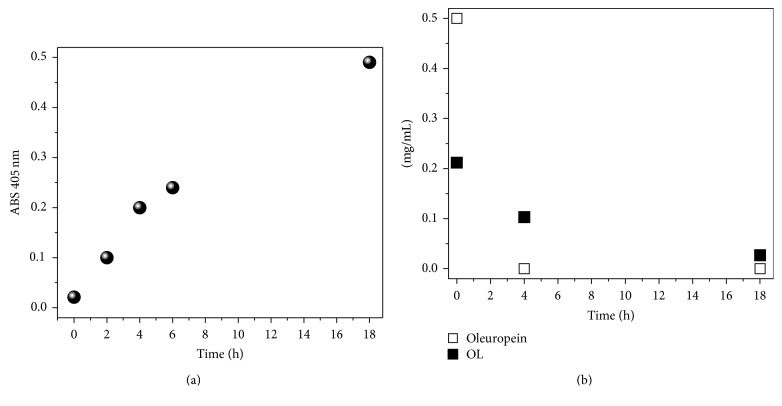
Changes of p-NPG measured by ABS at 405 nm (a) and reduction of oleuropein and olive leaf liquid complex (OL) amounts (b) performed in nutrient-poor medium (NWB-pH 5) by high cell concentration of the* L. plantarum* B11 strain.

This hypothesis was confirmed in a further experiment in which the* L. plantarum* B11 strain was tested separately towards the substrates p-NPG, oleuropein, and OL. Working at high cell concentration and without nutrients, the activity of the bacteria on the substrates was evident in the first hours, as Figure 5 shows. In Figure 5(a), *β*-glucosidase activity was explored by following the production of p-NP strictly related to the consumption of p-NPG as function of time. As shown, a substantial and constant *β*-glucosidase activity was established in the first 4 hours of the reaction. After six hours, reaction data appeared somewhat decreased and for longer time (18 hours) the enzymatic activity appears still considerable.

It should also be stated that olive fruit oleuropein degradation is different from pure oleuropein degradation since olive fruit properties can be affected by enzymatic activities and other different factors [7]. However, from this study we show that oleuropein degradation was complete after 4 hours, while 18 hours was needed to have a drastic consumption of OL substrate which, inter alia, contained also about 4% glucose (Figure 5(b)). Additionally, in both cases, the absence of both hydroxytyrosol and other hydrolyzed oleuropein derivates detectable by HPLC analysis was confirmed.

## 4. Conclusions

Characterization* in vitro* of two* L. plantarum* and one* L. pentosus* strains isolated from fermented green olive is described. All the strains showed interesting technological performance and good acidifying ability, highlighting their potential as possible starter for the production of fermented table olives. As regard to the oleuropein-degrading capability, divergent results emerged depending on the culture medium composition. Specifically, in nutrient-rich medium, a lower oleuropein-degrading action has been found. Conversely, oleuropein depletion in nutrient-poor medium was exhaustive and very fast, and it did not match with a significant production of hydroxytyrosol. Enzymatic profile assay has evidenced that all studied strains produced *β*-glucosidase activity but no esterase. From these results, it can be argued that microbial depletion of oleuropein could occur also through a biochemical mechanism different from hydrolysis. Nevertheless, microbial degradation of oleuropein could give different results in a real olive system. Thus, further* in vivo* investigations, based on the use of mono- or cocultures in olive fermentation, are necessary to confirm the existence of an alternative pathway for oleuropein degradation.

## Figures and Tables

**Table 1:** Identification of the isolated lactic acid bacteria based on blast comparison in GenBank. ^*∗*^Accession number of the sequence of the closest relative found by blast search.

Isolates	Size	Closest relative	% identity	Source^*∗*^
B3	643	*Lactobacillus plantarum*	99	DKJ917253.1
B4	642	*Lactobacillus pentosus*	100	JX129198.1
B11	642	*Lactobacillus plantarum*	99	KJ917253.1

**Table 2:** Api-Zym galleries of enzymatic activities corresponding to the *Lactobacillus *tested strains ((+) positive; (W) weakly positive; (−) negative).

Enzyme activities	*L. plantarum* B3	*L. pentosus* B4	*L. plantarum* B11
*β*-Glucuronidase	−	−	−
Alkaline phosphatase	−	−	−
Esterase (C4)	−	−	−
Esterase lipase (C8)	−	−	−
Lipase (C14)	−	−	−
Cystine arylamidase	−	−	−
Trypsin	−	−	−
*α*-Chymotrypsin	−	−	−
*α*-Galactosidase	−	−	−
*α*-Mannosidase	−	−	−
*α*-Fucosidase	−	−	−
Leucine arylamidase	+	+	+
Valine arylamidase	+	+	+
Naphthol-AS-BI-phosphohydrolase	+	+	+
*β*-Galactosidase	+	+	W
*α*-Glucosidase	+	+	+
*β*-Glucosidase	+	+	+
N-Acetyl-*β*-glucosaminidase	+	+	+
Acid phosphatase	W	W	W
